# Pseudogout: An Autoimmune Paraneoplastic Manifestation of Myelodysplastic Syndrome

**DOI:** 10.7759/cureus.3372

**Published:** 2018-09-27

**Authors:** Shumaila M Iqbal, Hafiz M Aslam, Faizan Faizee, Sana Qadir, Saadia Waheed

**Affiliations:** 1 Internal Medicine, University at Buffalo / Sisters of Charity Hospital, Buffalo, USA; 2 Internal Medicine, Hackensack Meridian School of Medicine at Seton Hall University, Nutley, USA; 3 Internal Medicine, Dow University of Health Sciences (DUHS), Karachi, PAK; 4 Internal Medicine, S & A Pediatrics, Parsippany, USA

**Keywords:** key words: myelodysplastic syndrome, pseudogout, autoimmune, paraneoplastic

## Abstract

Myelodysplastic syndrome (MDS) is often associated with autoimmune paraneoplastic manifestations. Seronegative arthritis is among one of them. Very rarely, pseudogout demonstrated as paraneoplastic autoimmune manifestations of MDS has been adumbrated so far. Our case would be the another addition in the series. Our patient is an 83-year-old male lately diagnosed with MDS. After six months of initial diagnosis, he had a sudden onset episode of pain and swelling involving left wrist. Synovial fluid analysis from respective radiocarpal joint confirmed the presence of intracellular positively birefringent rhomboid shaped crystals of calcium pyrophosphate dihydrate (CPPD). This was followed by another two flares of pseudogout involving right knee and lumbar spine at separate time intervals. Each of the episodes mentioned above responded well to intravenous and oral steroids. After the third bout, he was started treatment with azacitidine which showed effective abatement of further episodes of pseudogout up until now.

## Introduction

The accumulation of calcium pyrophosphate dihydrate (CPPD) crystals in the articular tissues leads to numerous manifestations including asymptomatic chondrocalcinosis, chronic CPPD inflammatory arthritis and acute CPPD crystal arthritis (pseudogout) [1]. The risk factors associated with pseudogout include hyperparathyroidism, hypothyroidism, hemochromatosis, electrolyte imbalances such as hypomagnesemia and hypophosphatemia, osteoarthritis, old age and prior joint injury [2]. Hereditary traits are also infrequently associated with pseudogout. Myelodysplastic syndrome (MDS) has been associated with autoimmune paraneoplastic manifestations, such as vasculitis, glomerulonephritis, hypothyroidism, inflammatory bowel disease, peripheral neuropathies, and seronegative inflammatory arthritis [3]. Majority of them have demonstrated an effectual response to steroid therapy [4, 5]. In the spectrum of seronegative inflammatory arthritides, as per authors’ knowledge, pseudogout’s association with MDS has been reported only a single time in the literature [1]. The reported case was found to be associated with complex karyotyping of MDS [1]. Discussed below is an anecdotal case of pseudogout manifesting as an autoimmune paraneoplastic sequela of MDS.

## Case presentation

**Figure 1:**
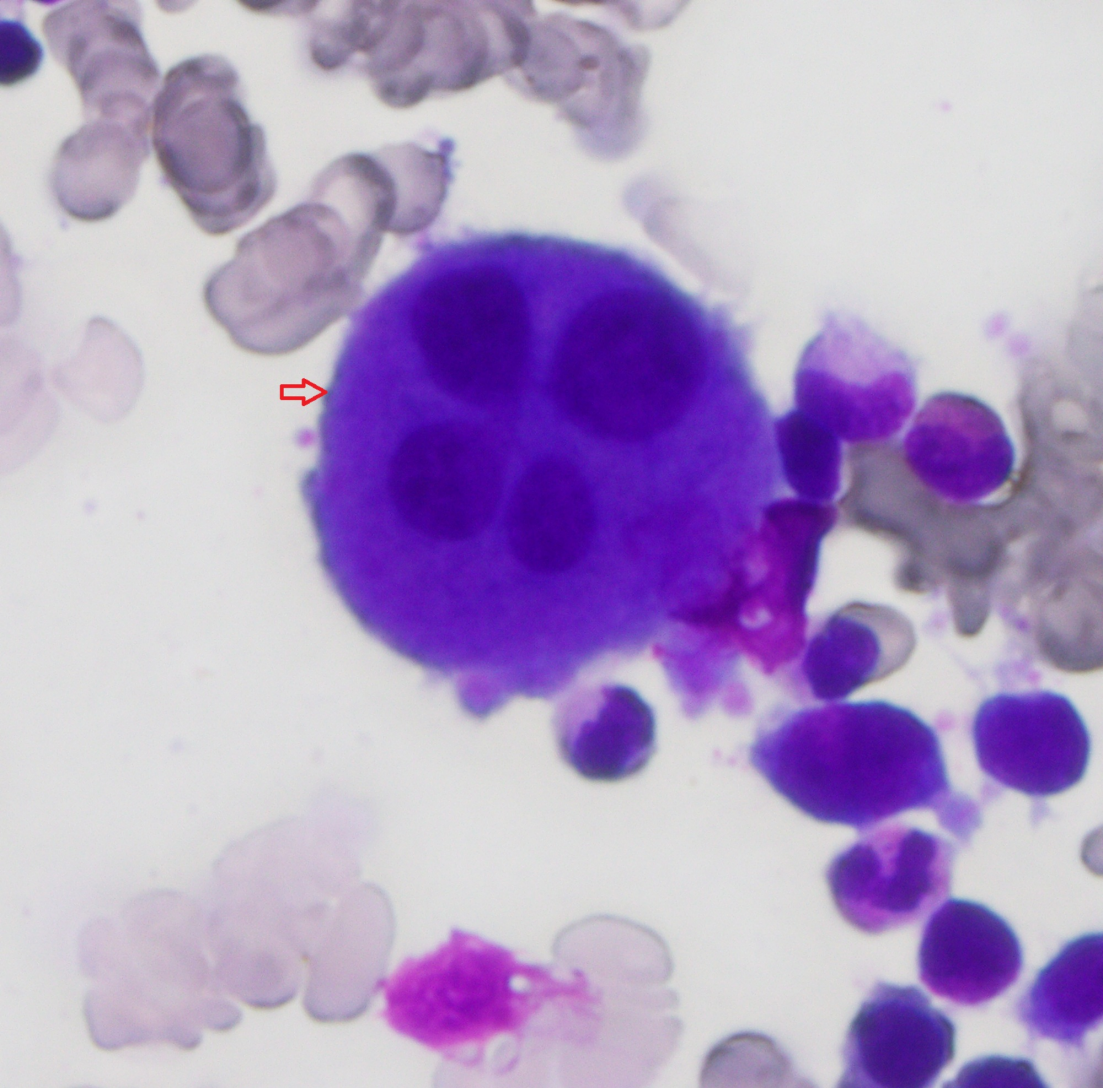
Bone marrow aspirant; arrow indicates dysplastic megakaryocyte.

**Figure 2:**
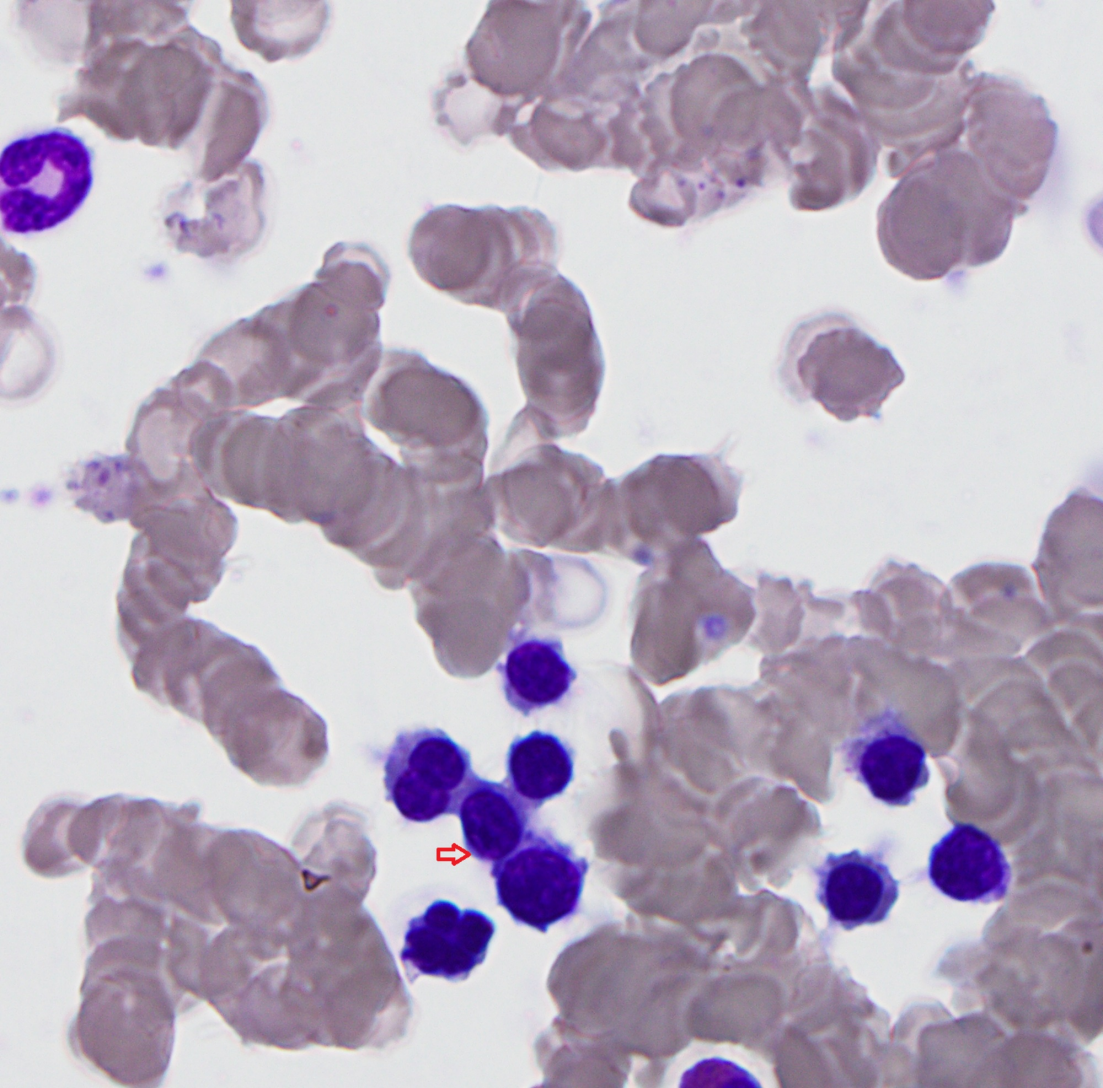
Bone marrow aspirant; arrow indicates dysplastic erythroids.

**Figure 3:**
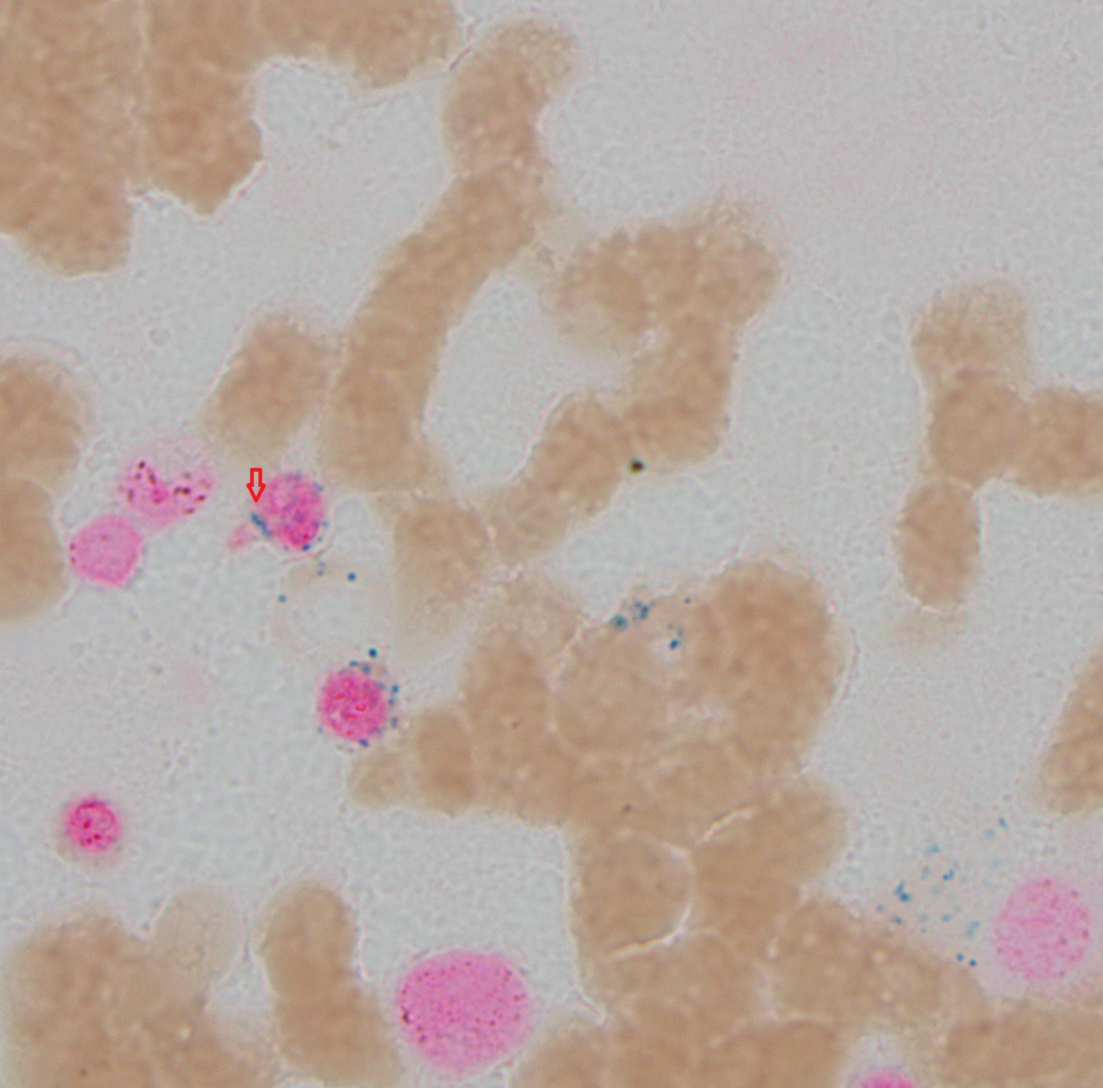
Bone marrow aspirant; arrow indicates ringed sideroblast which in turn is indicating dysplasia.

An 83-year-old male patient presented to the hematology-oncology clinic with the past medical history of small bowel obstruction status post resection, basal cell carcinoma of the skin, actinic keratosis and colon adenocarcinoma status post left hemicolectomy with negative postoperative surveillance for relapse by serial carcinoembryonic antigen levels and serial colonoscopies. He developed progressing pancytopenia. His laboratory workup revealed, hemoglobin 9.1 g/dl, platelet count 76,000/ul, white blood cell (WBC) count 2700/ul with 59% neutrophils, and absolute neutrophil count 1600/mm^3^. He had no reported past medical history of hematological disorders. No sign or symptom or any laboratory workup was indicative of systemic infection or inflammation. The patient’s home medications did not include any antimetabolite nor he was ever treated in the past with any antineoplastic agents or radiation therapy. The patient was a lifetime nonsmoker with occasional alcohol drinking. Vitamin B12 and folic acid levels were normal and HIV tests were negative, respectively. Bone marrow aspiration was performed which revealed myelodysplasia with ring sideroblasts (Figures 1-3). Cytogenetic results delineated complex abnormal karyotype with monosomy of chromosomes 5, 7, 20, partial deletion of 5q, and abnormalities consistent with high-grade myelodysplasia. The patient did not give consent for the proposed treatment with low dose chemotherapy. Thus management was begun with weekly intravenous (IV) erythropoietin administration along with as needed blood transfusion.

**Figure 4:**
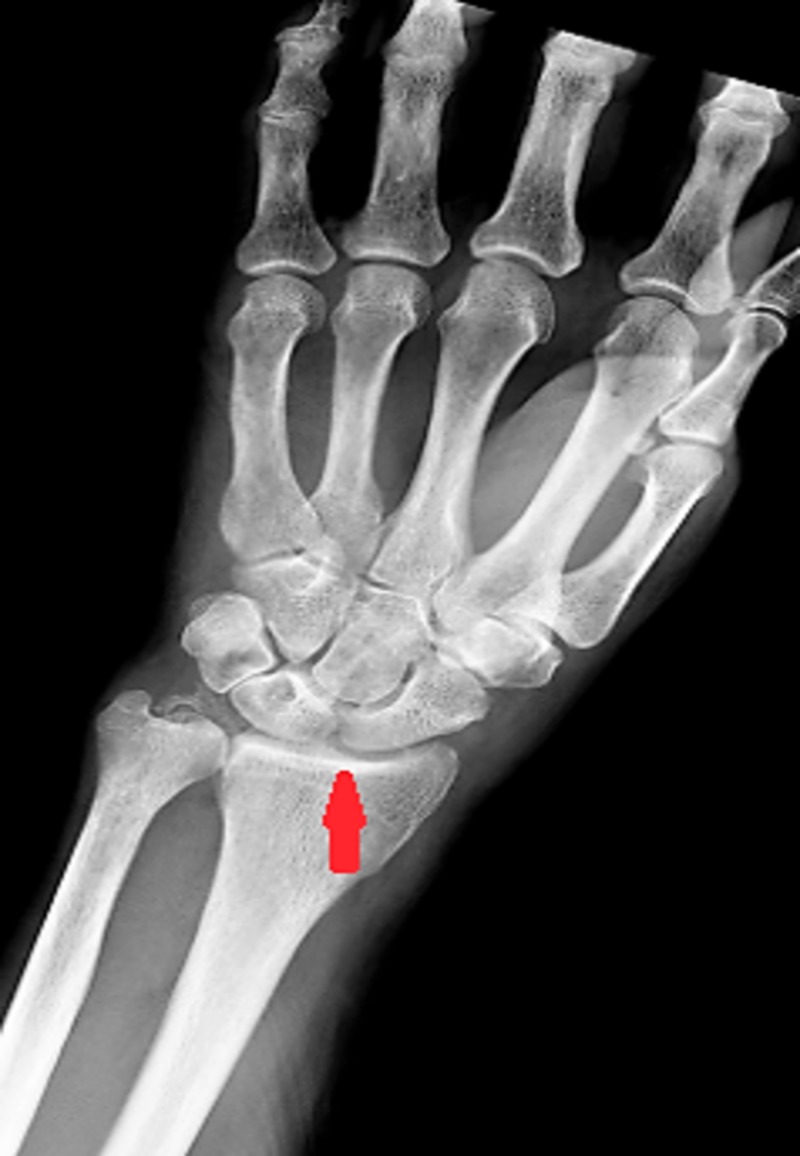
Chondrocalcinosis of radiocarpal joint.

Six months post-diagnosis, the patient was presented to the emergency department with the chief complaint of pain, swelling and redness of the left wrist-joint. He was afebrile and physical examination exhibited a swollen joint which elicited pain upon movement. X-ray of the wrist was evident for chondrocalcinosis (Figure 4). Erythrocyte sedimentation rate (ESR) and C-reactive protein (CRP) levels were elevated. Lab workup revealed WBC count 1.8 x 10⁹/L with 8% bands. Taking into account his leukopenia and inflamed wrist joint, the patient was empirically started on antibiotics for the clinical suspicion of septic arthritis but a minimal improvement in the symptoms was noticed. Synovial fluid extracted from the radiocarpal joint was grossly turbid with WBC count 6897/mm^3^ and polymorphonuclear granulocyte 87%. Gram staining was unremarkable and fluid cultures were negative for microbial growth. Crystal analysis of synovial fluid demonstrated few positively birefringent rhomboid intracellular CPPD crystals. Treatment with IV steroid was begun which culminated in a dramatic improvement of symptoms. Antibiotics administration was sustained due to a possibility of coexisting septic arthritis in the clinical context of leukopenia. The patient was discharged home on a steroid taper and a short duration course of antibiotics. Post-discharge, outpatient workup remained negative for antinuclear antibody, rheumatoid factor and cyclic citrullinated peptide antibody.

**Figure 5:**
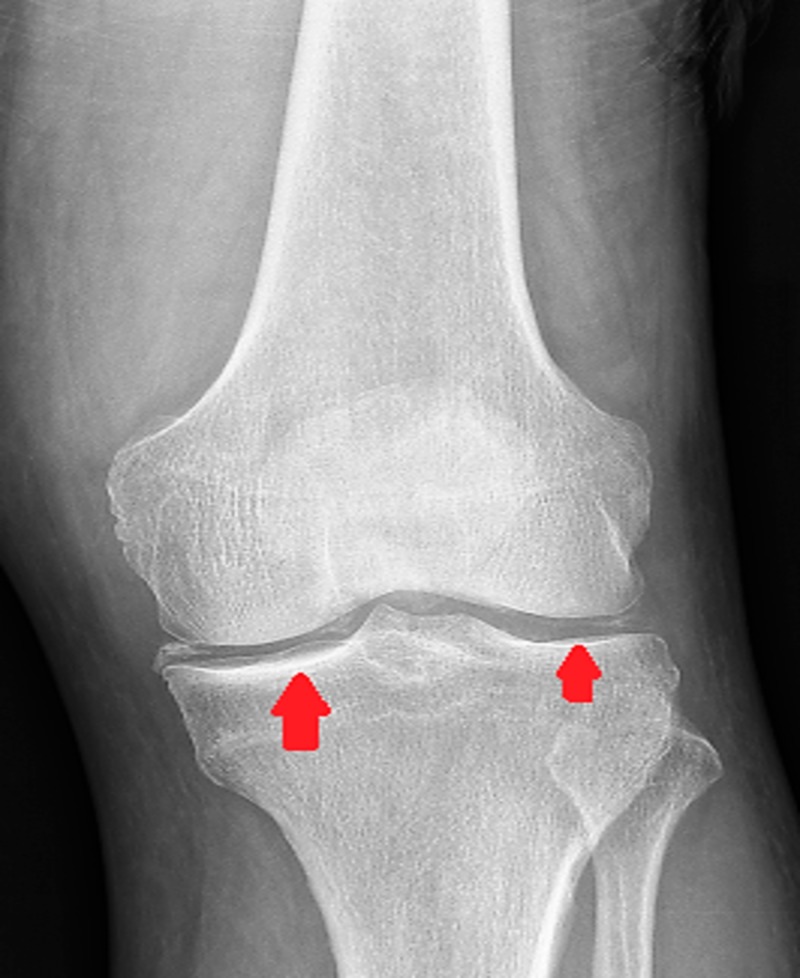
Chondrocalcinosis of knee joint.

A month later, the patient presented again with abrupt onset of right knee pain accompanied by joint swelling, tenderness and redness. X-ray indicated degenerative changes (chondrocalcinosis) of the knee joint most remarkable around the medial femorotibial compartment (Figure 5). Levels for ESR and CRP were elevated. Intracellular CPPD crystals were identified on synovial fluid analysis. The patient was treated with steroids and antibiotics (due to persistent leukopenia). The patient improves clinically. Colchicine could not be started for prophylaxis of pseudogout due to persistent leukopenia.

**Figure 6:**
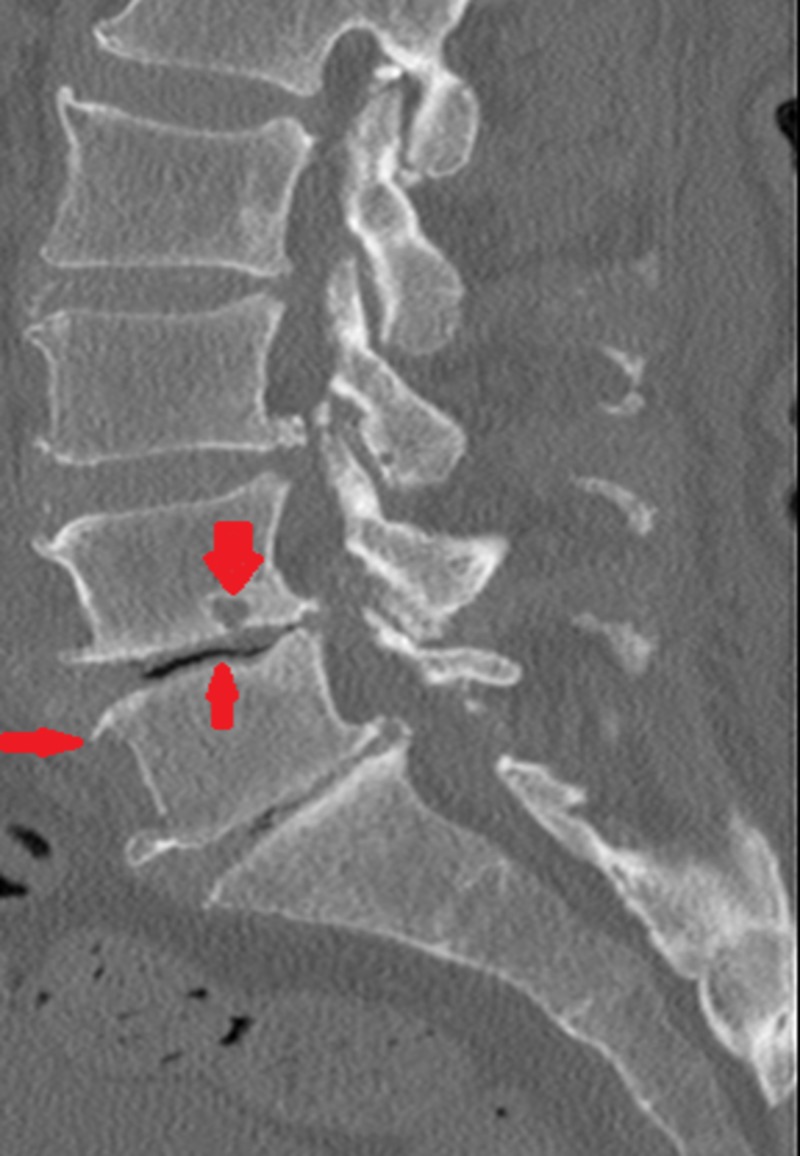
Degenerative changes at L4-L5.

A highly suspicious bout of pseudogout flared up again within a month with the symptoms of sharp lower back pain, localized to the lumbar area with redness surrounding overlying skin. The patient was afebrile. WBC count was 3500/ul. Degenerative changes were visible at L4-L5 on computed tomography (CT) spine with no contrast enhancing lesion identified on imaging (Figure 6). Considering history of previous flares of pseudogout, the patient was started treatment empirically with steroid in addition to antibiotics. Upon improvement, he was discharged home. Considering the evidence-based benefits of azacitidine in abeyance of the autoimmune phenomenon in MDS, the patient was then eventually started on azacitidine by his hematologist. No further acute CPPD crystal-associated acute flares of arthritis have been noted till date since the initiation of the therapy with azacitidine.

## Discussion

An intriguing case was reported in our hospital setting related to pseudogout associated with MDS. We found out a single case of this unique association in the medical literature [1]. Paraneoplastic syndrome is a term that describes a spectrum of clinical symptoms provoked by the production of different substances from a malignant neoplasm. The molecular mimicry portends by the released chemicals as well as the host immune response to the tumor cells results in various signs and symptoms [6]. The ubiquity of immune dysregulation in MDS characterized by an impaired CD8 response, an imbalance of T-regulatory cells and Th-17 cells also explain the inception of autoimmune manifestations [7]. At present, we do not have sufficient literature that could clarify the pathophysiological mechanisms behind the relationship of pseudogout with MDS. Hence, we hypothesize that our patient’s seronegative inflammatory arthritis shares the same phenomenon of immune aberrancy and impaired T-cell response like the other autoimmune manifestations (AIMs) of MDS [8].

Our patient’s arthritis did not convalesce with antibiotics as no organism was isolated from the synovial fluid tap; albeit, positively birefringent rhomboid intracellular calcium pyrophosphate crystals were detected in the synovial fluid which confirmed the diagnosis of pseudogout. Two causes, autoimmune paraneoplastic syndrome and administration of granulocyte colony-stimulating factor (G-CSF) in MDS patients for neutropenia [9, 10] are reported to manifest pseudogout in MDS, the former being a renowned cause. Because our patient’s multiple attacks of pseudogout involving several joints responded well to steroids, we speculated an autoimmune pathology behind it. Moreover, he was not administered G-CSF.

Cytogenetic abnormalities have been associated with AIM in MDS in nearly 50% of patients [11]. Numerous abnormal karyotypes have been recognised to provoke AIMs in MDS, however, 5-q deletions and monosomy 7 are the most commonly identified. Seronegative inflammatory arthritis, an AIM of MDS, is specifically found to be associated with more complex karyotyping [12]. The intricacy of cytogenetic abnormality is a prime tool in postulating the prognosis of AIM in MDS [11]. Deletions within the long arm (q) on chromosome 5 cause sustained NF-kappa B signalling which results in activation of genes regulating inflammatory responses and apoptosis [13]. MDS-related arthritis involves multiple asymmetrical joints in a migratory fashion with infrequent association with positive Rh factor. Thus, autoimmune seronegative inflammatory arthritis is a classic type of arthritis observed in MDS patients [5, 7]. The precise mechanism of AIM in MDS remains elusive and requires extensive clinical data. Many clinical analyses have revealed that treating the intrinsic tumors can control both the progression of the tumor and its related AIMs [14]. Azacitidine and Decitabine have been documented to be beneficial in MDS; numerous evidence has conferred an exceptional response of bone marrow to treatment as well as remission of autoimmune symptoms [15]. Since we initiated our patient on azacitidine by taking into account its advantages, symptoms of autoimmunity or joints pain were reduced.

## Conclusions

Myelodysplastic syndrome patients have been repeatedly reported with various AIM including seronegative inflammatory arthritis. Pseudogout should be suspected as one such possibility in context of seronegative inflammatory arthritis in patients with MDS. Since abnormal T-cell response is involved in the induction of autoimmune traits, steroid therapy serves as a cornerstone in subduing autoimmune flare of pseudogout in MDS. Moreover, MDS and its relevant paraneoplastic inflammation can adequately be rectified with Azacitidine. In this modern epoch of medicine, we foster the evolution of genuine therapeutic strategies for MDS-associated inflammatory arthritis.
